# On the Constitutional Effects Prodnced by Substances Introduced into the Urethra

**Published:** 1858-06

**Authors:** I. L. Crawcour

**Affiliations:** Professor of Chemistry and Medical Jurispurdence, New Orleans School of Medicine


*On the Constitutional Effects produced by Substances introduced into the Urethra.* By I. L. CRAWCOUR, M. D., F. S. A., Professor of Chemistry and Medical Jurispurdence, New Orleans School of Medicine.

In the summer of 1857 I noticed that when bougies, tipped with an ointment containing morphine, were introduced into the urethra, rapid and intense narcotism was produced; and so intense and constant is the phenomenon, and so out of all proportion to the quantity of sedative employed, that I deem it my duty to lay before the profession this remarkable circumstance, especially as I can find no mention of it in books, and as even in some, the absorbing property of the mucous lining of the urethra and bladder is denied. In April, 1857, having a patient afflicted with chronic inflammation of the urethra, and who suffered intense agony whenever an instrument was passed, I thought relief might be obtained if, instead of using oil, I smeared the instrument with morphine cerate. Accordingly I prepared an ointment containing twenty grains of morphine in every drachm of spermaceti ointment, and as the inflammation was confined to the membranous and prostatic portions of the canal, I smeared the last three inches of the instrument (a silver sound) only, with ointment, passed it and allowed it to remain about five minutes.—The patient complained of feeling very sleepy, and the next day stated that he had hardly been able to walk home, and as soon as he arrived there, fell into a profound slumber. Thinking this was simply a coincidence, I again applied the ointment in the same manner and a similar result ensued. Desirous of testing whether this was not an idiosyncrasy on the part of my patient, I passed a bougie, prepared in a similar maner, on myself, and to my surprise it had not been in my urethra three minutes before all the symptoms ordinarily produced by opium developed themselves.

On the twenty-third of May another opportunity presented itself for testing this singular property of the urethral membrane in a most striking manner, on a patient whom I will call A. I passed a bougie tipped with morphine ointment, consisting of a drachm of morphine to an equal quantity of cerate, and immediately after passed another smeared with an ointment consisting of ten grains of atropine to the drachm of cerate.—The patient almost immediately, that is in less than a minute, became incapable of locomotion. I left him asleep in my office at nine o'clock, and on returning at two I found him still asleep and vomiting had taken place. It was not until four o'clock that he was capable of walking home, and then he staggered like a drunken man, and complained of a numb sensation over the whole body. Two days after, he called on me and said that as he went home he heard many persons say he was drunk, but was unable to speak—was unable to answer any question when he reached his boarding-house; as soon as he arrived there, went to bed and did not awake until twelve o'clock the next day. He says he still feels under the influence of the drug.

A few days after this, another patient presented himself—a strong, robust healthy man, with a remarkable muscular development. I passed a catheter tipped with a minute quantity of the atropine ointment, certainly not as much would be equal in size to a pin head; the instrument remained in about five minutes; absorption was very rapid; even while it was in, I noticed that the pupils dilated and the voice became husky; in ten minutes, when about leaving my office, he complained of feeling drunk and numb, and walked with staggering gait. He called on me the next day and said that when he got home he felt perfectly drunk, was unable to sleep, had constant confusion, sense of giddiness, inability to speak, his throat felt dry and parched, was unable to see. This morning he feels perfectly recovered.

On the 28th of May I passed a silver sound on Mr. H., tipped with the morphine cerate. Drowsiness was produced in five minutes. He called on me the next morning, at half-past nine, to evacuate his bladder—paralysis of the bladder having occurred and retention since half-past three of the previous day, the period when the instrument was passed.

During the past winter one of our students, who suffered from stricture of the urethra, came to me for relief. As he was a very intelligent young man I considered this a very fair case for experiment, and having explained to him the nature of what I was about to do, obtained his consent to pass a bougie tipped with atropine, with the promise that he would analyze his sensations and furnish me with a written account of what he experienced. I append in his own words the result of the experiment: "On arising from the couch on which I was resting I experienced a sensation of giddiness in the head, with impaired vision, which passed off in a few minutes. These symptoms were accompanied by dilatation of the pupil. After walking a short distance these sensations returned and my gate became very unsteady. Dryness of the mouth and fauces now occurred, the secretion of saliva became almost entirely suspended, so much so, that the use of tobacco would not reproduce it. These phenomena were followed by an intense desire to urinate with inability to do so. In this condition I remained six or eight hours, when I succeeded in getting to sleep. On awaking the following morning these effects had very nearly subsided, a little dryness of the fauces being all that remained, and this gradually passed off durimg the day." Such were the sensations produced on this gentleman by an almost infinitessimal quantity of the alkaloid applied in the manner I have indicated, and the phenomena commenced in five minutes.

Since this I have employed bougies smeared with morphine and atropine ointment in at least fifty cases, and in every instance the result has been the same; but as I was desirous that this singular phenomenon should be witnessed by some of the profession, I obtained permission from Dr. Choppin to pass a boagie, similarly prepared, on a patient in his ward, in the Charity Hospital. The patient was a man not suffering from any disease of the urethra, and I passed a No. 7 silver sound, smeared at its extremity with atropine ointment, into his urethra, in the presence of Dr. Choppin. Within three minutes from its introduction, as witnessed by Dr. Choppin and several students, the pupils dilated completely; in five minutes the phantasmal delirium, so characteristic of belladonna, was developed to such an intense degree as to necessitate mechanical re-traint. All these effects were produced by a quantity of atropine so minute as to be almost inappreciable. The strength of the oint-men was ten grains to the drachm; the quantity smeared on the bougie did not weigh half a grain, equaling the twelfth of a grain of atropine. On withdrawing the instrument, apparently the whole of the ointment was adhereing to it, and it is probable that not the fiftieth of a grain of ointment had been removed or absorbed. This would reduce the quantity of atropine to an almost infinitessimal quantity, and I have not the slightest doubt that certainly less than the three-hundredth of a grain entered the system. But allowing even that the whole quantity absorb-ed, it would still be inexplicable how absorption should be so rapid and the effects produced so powerful. The usual dose of atropine is about the twentieth of a grain, of morphine from the sixth to one grain; yet in no instances, administered either by the stomach or endermically, would their effects be comparable to those I have seen and just describ-ed, as when applied to the urethral mucous membrane.

There is also another circumstance I have observed, viz.: that this absorbing power is not common to the whole urinary tract. The bladder and the spongy portion of the urethra seem incapable of absorbing. I have injected three drachms of Battley's sedative into the bladder and allowed it to remain there, without perceiving the slightest general influence. At the same time, if we pass a prepared bougie only four or five inches into the urethra, we shall not perceive any narcotic influence however long it may remain there; pass it an inch or inch and half further, and ripid narcotism ensues. It seems as if the prostatic portion of the urethra and the neck of the bladder were the only portions possessed of this absorbent property, and that the rest of the canal was inert. In order to test this, I injected into my own urethra a saturated solution of morphine, and held it for some ten minutes—of course it could not penetrate beyond the membranous portion—not the slightest narcotic effect was produced. The next day I passed also, upon myself, a bougie, smeared at its distal extremity with morphine, to the neck of the bladder, and in three minutes absorption took place, accompanied by all the effects of a full dose of opium. If we take a bougie with its last two inches only, smeared with morphine, and pass it *rapidly* into the bladder, allowing its extremity to rest aginst its walls, but its coated portion being beyond the prostate and neck, no absorption takes place, however long it may remain there; withdraw it two inches, and now allow the morphine to come in contact with the neck, absorption again rapidly ensues. These circumstances, therefore, conclusively point to the fact, that this elective power resides only in a small portion of the urinary membrane—a portion bounded by the prostate and neck of the bladder—there is a possibility that the trigone may have some absorbing power, although this is doubtful.

With regard to the rationale of this curious phenomenon, I am at a loss—it is true that the part where I have located absorption, is the one most richly endowed with nerves and blood vessels--the hypogastric plexus of the sympathetic ramifying over the prostatic urethra and neck of the bladder. Here they are very numerous and of a large size, and according to Valentin and Muller, have ganglionic swelling on various points. We have also a large congeries of blood vessels at this point, but the absorption and intensity of effects are so out of all proportion to what we observe when sedative are applied to other organs, that I cannot hazard a theory. I have simply stated the facts as I have observed them, and shall leave it to others to frame a hypothesis to account for them. I would suggest that this property, which I have indicated mignt be found useful in certain cases of mania and dilirium tremens, where we wish to get patient under the influence of a narcotic, and where they refuse to swallow. In these cases we can always pass a bougie smeared with atropine or morphine into the urethra, and I am convinced that speedy and full narcotism would be the result.—*N. O. Med. News and Hospita Gazette.*

